# Should General Practitioners Issue a Sick Certificate to Employees Who Consult for Low Back Pain in Primary Care?

**DOI:** 10.1007/s10926-014-9564-z

**Published:** 2015-01-17

**Authors:** M. Lewis, G. Wynne-Jones, P. Barton, D. G. T. Whitehurst, S. Wathall, N. E. Foster, E. M. Hay, D. van der Windt

**Affiliations:** 1 Arthritis Research UK Primary Care Centre, Institute of Primary Care and Health Sciences, Keele University, Staffordshire, ST5 5BG UK; 2 Health Economics, School of Health and Population Sciences, College of Medical and Dental Sciences, University of Birmingham, Edgbaston, Birmingham, B15 2TT UK; 3 Faculty of Health Sciences, Simon Fraser University, 8888 University Drive, Burnaby, BC V5A 1S6 Canada; 4 Centre for Clinical Epidemiology and Evaluation, Vancouver Coastal Health Research Institute, 828 West 10th Avenue, Vancouver, BC V5Z 1M9 Canada

**Keywords:** Back pain, General practice, Certification, Clinical effectiveness, Cost effectiveness

## Abstract

*Purpose* Back pain is a common problem and has significant societal impact. Sickness certification is commonly issued to patients consulting their general practitioner with low back pain. The aim of this study was to investigate the association of certification for low back pain with clinical outcomes and cost consequences. *Methods* A prospective cohort study using linked questionnaire and medical record data from 806 low back pain patients in 8 UK general practices: comparison of 116 (14.4 %) who received a sickness certificate versus 690 who did not receive certification. The primary clinical measure was the Roland and Morris Disability Questionnaire (RMDQ). Data on back pain consultation and work absenteeism were used to calculate healthcare and societal costs. *Results* Participants issued a sickness certificate had higher back-related disability at baseline consultation and 6-month follow-up [mean difference 3.1 (95 % CI 1.8, 4.4) on the RMDQ], indicating worse health status. After fully adjusting for baseline differences, most changes in clinical outcomes at 6 months were not significantly different between study groups. Productivity losses were significantly higher for the certification group, with most absence occurring after the expected end of certification; mean difference in costs due to absenteeism over 6 months was £1,956 (95 % CI £941, £3040). *Conclusions* There was no clear evidence of a difference in clinical outcomes between individuals issued a sickness certificate and those not issued a certification for their back pain. With little overall contrast in clinical outcomes, policy makers and care providers may wish to draw on the likely difference in societal costs alongside issues in ethical and moral care in their consideration of patient care for low back pain.

## Introduction

Back pain is common in the workplace and the community at large, and its disabling characteristics render it a significant burden to society. In the United Kingdom (UK), the 1-month prevalence of low back pain in the adult general population (pain lasting >1 week) has been estimated to be 23 % [1]. One in seven consultations in primary care is for musculoskeletal pain, with back pain being the most common reason for consultation at an annual rate of 591 people per 10,000 registered persons [2].

It is well known that back pain has a major impact on productivity at work. In the UK, annual costs of back pain have been estimated as £1.6 billion for direct healthcare costs (including consultations with healthcare professionals, hospital outpatient visits and in-patient days) and £10.7 billion for indirect factors (including informal care and workplace productivity costs) [3]. In most countries a sickness certificate sanctions absence from work, although issuing requirements differ between countries. In the UK, a sickness certificate is required for periods of work absence in excess of 7 days [4].

Musculoskeletal disorders and mental disorders are the most frequent reasons for sickness certification [4–7], and the most common causes of long-term work absence [8]. In the UK, approximately one-third of back pain general practice consulters are issued a sickness certificate [9]. Aside from workplace factors little is known about the comparative clinical features of patients who are issued a sickness certificate compared to those who are not: conceivably patients issued a certificate have greater physical impairment that interferes with their ability to perform usual work activities. Despite the high rates of sickness certification and possible rationale for alleviating work difficulties through sickness certification, recent research suggests that it may not be clinically effective [10], and several studies have shown that sickness absence and certification is associated with increased risk of long-term disability and subsequent award of a disability pension [11–16]. There is little data contemporaneously comparing the clinical and cost outcomes of patients who receive a sickness certificate with those who do not.

In light of this evidence on high costs, particularly through workplace productivity loss, the UK government commissioned health and work as a key priority target for public policy, setting recommendation and guidelines for the provision of alternative/altered work duties for workers unable to perform their usual job activities [17–19]. Although plausible that this strategy will lead to reduced cost to society, there is no clear data or evidence to date to steer primary care policy makers and providers as to whether the strategy would be associated with improved clinical outcomes or reduced healthcare or societal costs.

Current recommendations about care of low back pain in primary care encourage patients to stay active (including work), and highlight the importance of self-management, whilst advocating the use of the Back Book for simple clinical guidelines [20, 21]. However, Bishop et al. [22] showed that current attitudes and behaviours of general practitioners and physiotherapists towards patients with low back pain are diverse, and that many practitioners wrongly held the belief that patients should avoid activities and work. Gerner and Alexanderson [23] reported that physicians were often faced with difficult and distressing decisions with regards to sickness certification, and that potential discordance in doctor–patient opinions was highly likely since sickness certification legislation is based on impaired work ability by assessment. In all, Soler and Okkes [24] asserted that sick certification was “an unwelcome administrative burden for the family doctor”. It could be argued that this burden and contention in its use needs to be balanced against ethical considerations in relation to such issues as the need to: express empathy and provide compassionate care, reduce unnecessary pain and suffering, prevent an exacerbation of symptoms, or to avoid embarrassment and stigmatization at work.

This study aimed to; (1) compare clinical and demographic characteristics between patients issued a sickness certificate by their general practitioner (GP) for their low back pain and those who are not issued a certificate, (2) evaluate whether or not issuing sickness certificates is associated with clinical outcomes, and (3) estimate the cost consequences associated with sickness certification.

## Materials and Methods

### Study Population

This study is based on a longitudinal dataset describing the characteristics and clinical outcomes of patients from the Beliefs about Back Pain (BeBack) cohort [25]; a prospective observational cohort study that recruited consecutive back pain consulters receiving usual primary care, aged 18–60 years, from eight GP practices in North Staffordshire and Cheshire. The GP practices cover a heterogeneous socio-demographic population mix in terms of urban/rural profiles. The BeBack study used mixed-methods to investigate patients’ illness perceptions and psychological obstacles to recovery in relation to back pain [25, 26], and investigate health care professionals’ (general practitioners and physiotherapists) beliefs and attitudes about low back pain [27, 28].

Patient participants were identified if they consulted their GP for low back pain. Recruitment of participants was by weekly downloads of back pain diagnostic codes [29], from the computerised system of the eight general practices. Eligibility was based on primary care consultation for non-specific low back pain (i.e. excluding ‘red-flags’ indicative of possible serious spinal pathology e.g. cancer, ankylosing spondylitis, cauda equine syndrome, significant trauma) inclusive of acute, sub-acute and chronic pain. Downloaded lists were checked for suitability by the GPs concerned. In the UK, coding of morbidities by GPs follows the electronic Read code classification system; this system has been validated [30–32]. Broad morbidities, including back problems, are usually linked to several Read codes—to reflect different forms and symptoms of disease. The coding system is hierarchical to reflect the multi-layered pathway to diagnosis. Low back pain, particularly, has a diverse set of Read codes: The following Read codes were used to identify consultations logged for back pain: 16C2-16C9; 16CA; 16CZ; N140-1; N1402; N142; N142-1/3/4; N1420; N143; N143-1; N145; N145-1/2; S57z(0).

A study pack was sent from the patients’ GP practice to each potential participant in the week following consultation. Non-responders were sent a reminder postcard after 2 weeks; if necessary, a further questionnaire was sent after 4 weeks. Patients were recruited between September 2004 and April 2006, with follow-up at 6 months. For those participants who completed the baseline questionnaire, reported being employed (for the employment status question in the questionnaire), and who gave consent to medical record review, the electronic records of their sickness certificates were downloaded to identify those with and without a sickness certificate. The date of questionnaire return was used as a reference and sickness certificates issued in the month prior to this date matched to individual patients. Since the baseline mailing process was initiated for each individual in the week following consultation with the GP and to allow for mailing response delay it was decided that we would adequately capture the associated GP-consultation and healthcare utilisation and sickness certification data relating to the back pain consultation if we targeted the medical record review as 31 days prior to baseline response. This method of data retrieval has been demonstrated to link 95 % of sickness certification records with self-reported absence for an episode of back pain [33]. Hence, the total time period of the study is 31 days prior to baseline response through to 6 months follow up (post baseline response) with healthcare utilisation being measured within this 7 months total time-frame.

### Comparison Groups

Two groups of patients were defined for comparison:

1. Sick certification (SC) group—Patients who did receive a sickness certificate during the 31 days prior to the completion of baseline survey.
2. No sick certification (N-SC) group—Patients who did not receive a sickness certificate during 31 days prior to the completion of the baseline survey.

### Outcome Measures

The main clinical outcome measure was back-specific functional disability measured using the 24-item Roland and Morris Disability Questionnaire (RMDQ) [34], with a score ranging from 0 (no back pain-related disability) to 24 (highest back pain-related disability). A difference of 2.5 points on the RMDQ scale is considered a minimal clinically important difference [35]. Secondary self-report outcomes included measures of pain severity, quality of life and psychological consequences of pain: Chronic pain grade (CPG), including subscales measuring interference with normal functioning and work (using two 0–10 numerical rating scales) [36]; pain intensity (“today” and “over the past 2 weeks”, using 0–10 point numerical rating scales); bothersomeness of pain in last 2 weeks (5-point ordinal scale) [29]; preference-based health-related quality of life (EQ-5D) [37]; anxiety and depression (Hospital Anxiety and Depression Scale, HADS) [38]; fear of movement (Tampa Scale of Kinesiophobia, TSK) [39]; catastrophising (subscale of the Coping Strategies Questionnaire, CSQ) [40], and pain self-efficacy (pain self-efficacy scale PSES) [41]. Higher scores on the numerical scales indicate worse symptoms/perceptions/interference, except for the pain self-efficacy scale and EQ-5D (where higher values denote better health status).

### Statistical Analysis for Outcomes

Differences between groups at baseline were assessed using the independent samples *t* test (for numerical measures) and Chi squared test (for categorical measures) with statistical significance at the 5 % two-tailed level. Between-group mean differences in outcomes at follow-up were evaluated through linear regression. We performed a sequential adjustment on estimates of health outcomes to identify potential confounding with respect to: (1) baseline socio-demographic covariates only [age, gender, socio-economic class, GP Practice]; and (2) baseline socio-demographic and pain/disability covariates [socio-demographic plus RMDQ and pain-scales]; (3) baseline socio-demographic and pain/disability and psychosocial and general health covariates [socio-demographic and pain/disability covariates plus CPG; bothersomeness, EQ-5D; HADS-A; HADS-D; TSK; CSQ and PSES].

### Resource Utilisation and Costs

Two cost perspectives were considered: (1) a health care perspective, which includes health care resource use in primary and secondary care, and (2) a broader perspective that incorporates costs associated with lost workplace productivity due to absenteeism.

The estimation of healthcare costs was based on resource use data from participants’ medical records collected between 1 month (31 days) prior and 6 months (180 days) after the completion date of the baseline questionnaire. The cost estimation exercise included: all consultations with healthcare professionals, referrals to secondary care and allied health professionals, and days of work absence. Costs were attached to each resource entry in accordance with the unit cost sources outlined in Table 1, which reflect national average valuations in 2005.

**Table 1:** Unit costs of resources based on 2005 UK prices

Resource	Source	Cost (£)
*Primary care/community care consultations*		
GP		
Surgery	Curtis and Netten (S9.8b)	24 (10 min. consultation)
Telephone	Curtis and Netten (S9.8b)	24 (10.8 min. consultation)
Home	Curtis and Netten (S9.8b)	69 (inclusive of travel time)
Practice nurse	Curtis and Netten (S9.6)	10
Nurse practitioner	Curtis and Netten (S9.7)	15
Physiotherapist	Curtis and Netten (S8.1)	20^a^
Health care social worker	Curtis and Netten (S10.2)	31
*Secondary care referral costs*		
Orthopaedic surgeon—consult	Curtis and Netten (S14.5)	108
Orthopaedic surgeon—admission	NHS Exec. Code R02	3,343
Rheumatologist	Curtis and Netten (S14.4)	107
Hospital physiotherapist	Curtis and Netten (S12.1)	15^a^ (per 20 min. session)
Radiographer	Curtis and Netten (S12.5)	18
*Private referrals and to alternative care*		
Orthopaedic surgeon	As for NHS orthopaedic consultation	108
Physiotherapist	As for NHS physiotherapist	15^a^ (per 20 min. session)
Chiropractor	College of Chiropractors	35^a^
*Indirect costs*		
Based on average hourly wage within social economic class	ONS—Annual Survey of Hours and Earnings (ASHE)	Range 6.21–21.77

^a^Number of sessions costed for is 4 based on previous studies showing average number of therapist sessions [33]

Data on indirect costs were ascertained by linking estimates of lost work time from information in the 6-month follow up self-report questionnaire and sick-certification medical record data with national salary estimates. Participant’s current job titles were sought at baseline and these job descriptions were coded according to the Standard Occupational Classification (SOC: 2000) [42]: salary estimates were attached to each of these codes based on ONS national survey valuations [43]. At 6 months follow-up, productivity loss was established by enquiring about time off work (absenteeism) in the previous 6 months. Productivity cost was calculated as the product of the number of days off work and the average daily wage.

Costs were averaged across all individuals in the two groups and represent approximately half-year costs (not annualised costs). Differences in mean outcomes and mean costs were compared between the two study groups: the N-SC group being used as the reference group in the analyses. Cost data in health care research is, typically, positively skewed. Accordingly, bias-corrected and accelerated bootstrapping (BCa) was used to derive confidence intervals for cost estimates (1,000 replications) [44, 45].

### Sensitivity Analysis

The main approach to calculating productivity costs was through the human capital approach (HCP) whereby costs were calculated over the full period of absenteeism. As a sensitivity analysis we also calculated productivity costs using the friction cost approach (FCA) with a valid friction period of 3 months [46].

### Ethical Approval

Ethical approval for the BeBack study was granted by North Staffordshire LREC, reference number 04/16.

## Results

**Fig. 1:**
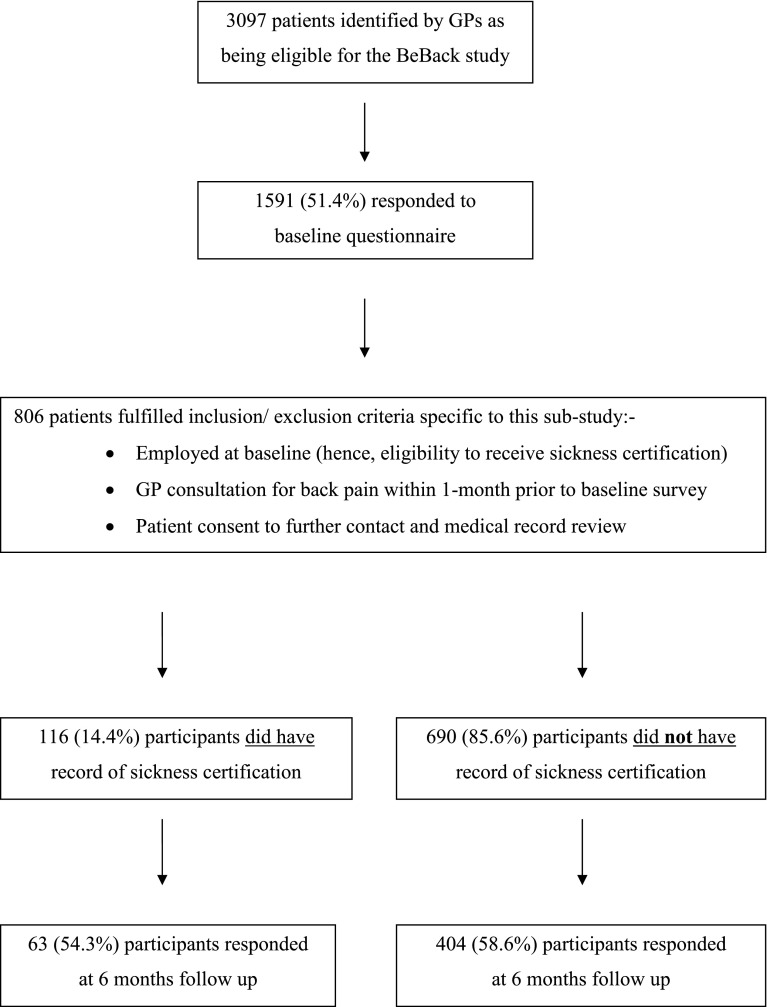
Flowchart of recruitment into the study and participant follow-up

3,097 patients were invited to participate in the cohort study, meeting the inclusion criteria of consulting their GP with an episode of low back pain, 1,591 (51.5 %) completed the baseline questionnaires, 1,289 (80 %) gave consent for further contact and medical record review. 806 patients fulfilled the additional inclusion/exclusion criteria related to this sub-study. 467 (57.9 %) completed the follow-up questionnaire at 6 months. Of these 806 study participants, a total of 116 (14.4 %) patients received a sickness certificate in the month prior to completion of their baseline survey (SC group); 690 did not receive a sickness certificate for back pain (based on the codes for certification used in our medical record search) (N-SC group). Response rates at 6 months follow-up were similar in the SC group (n = 63, 54 %) and N-SC group (n = 404, 59 %). A flowchart illustrating recruitment and follow-up for this sub-study is shown in Fig. 1.

### Outcomes

The SC group had higher mean baseline scores for back-related disability (RMDQ), pain-measures, anxiety and depression (HADS) and fear of movement (TSK), and lower scores for health-related quality of life (EQ-5D) and pain self-efficacy—all indicating worse health status. The SC group were also more likely to be Grade IV on the CPG (reflecting chronic/severe pain) and to be ‘very’ or ‘extremely’ bothered by their low back pain (see Table 2).

**Table 2:** Baseline characteristics of the sickness certification (SC) and non-certification (N-SC) groups

	SC group n = 116	N-SC group n = 690
Age		
Mean (SD)	42.1 (9.8)	43.4 (9.9)
Gender*		
Females	52 (44.8 %)	384 (55.7 %)
Males	64 (55.2 %)	306 (44.3 %)
Occupational class*		
Non-manual	43 (37 %)	370 (54 %)
Manual	73 (63 %)	320 (46 %)
Roland and Morris Disability Questionnaire*		
Mean (SD)	11.6 (5.8)	7.4 (5.4)
Usual pain—past 2 weeks*		
Mean (SD)	5.4 (2.6)	4.1 (2.5)
Pain today*		
Mean (SD)	4.7 (2.7)	3.5 (2.6)
Duration of pain		
<1 month	39 (34.8 %)	292 (44.0 %)
1–3 months	34 (39.3 %)	203 (30.6 %)
4–6 months	14 (12.5 %)	56 (8.4 %)
7 months—3 years	11 (9.8 %)	58 (8.7 %)
>3 years	4 (3.6 %)	54 (8.1 %)
Chronic pain grade*		
I	15 (12.9 %)	213 (31.3 %)
II	7 (6.0 %)	182 (26.7 %)
III	38 (32.8 %)	176 (25.8 %)
IV	56 (48.3 %)	110 (16.2 %)
Bothersomeness*		
Not very much	37 (31.8 %)	364 (53.3 %)
Very much	79 (68.2 %)	318 (46.7 %)
EQ-5D*		
Mean (SD)	0.55 (0.27)	0.69 (0.24)
Hospital Anxiety and Depression Score—anxiety*		
Mean (SD)	8.6 (4.0)	7.7 (4.3)
Hospital Anxiety and Depression Score—depression*		
Mean (SD)	7.6 (3.9)	5.7 (3.9)
Tampa Scale of Kinesiophobia*		
Mean (SD)	41.5 (6.1)	38.9 (6.7)
Catastrophising Scale		
Mean (SD)	11.0 (7.9)	8.7 (7.2)
Pain self-efficacy scale*		
Mean (SD)	31.0 (13.4)	41.6 (12.8)
Satisfied with work*		
Mean (SD)	31.0 (13.4)	41.6 (12.8)

Figures are frequency counts (column percentages) unless otherwise specified

* *P* < 0.05 (by *t* test for numerical variables, Chi square test for categorical variables (Chi square test for linear trend for ordinal variables)

*I* low disability–low intensity, *II* low disability–high intensity, *III* high disability-moderately limiting, *IV* high disability-severely limiting

‘Not very much’ = ‘not at all’ or ‘slightly’ or ‘moderately’; ‘Very much’ = ‘very much’ or ‘extremely’

At 6 months follow-up several of the mean differences in health outcomes remained statistically significant in the direction of worse health status for the SC group (see Table 3). The primary measure, the RMDQ, was 3.1 points (95 % CI 1.8, 4.4) higher at 6 months; SC group 7.3 (SD 6.2) versus N-SC group 4.2 (SD 4.6). There was also significantly greater interference with work and usual daily activities for the SC group. Associations with clinical outcomes at 6 months were still statistically significant and changed little when adjusting for baseline differences in socio-demographic variables. However, all associations (but particularly pain and function outcomes which were no longer statistically significant) were considerably weakened when further adjusting for baseline pain and disability scores. Additional adjustment for baseline psychosocial and general health variables further reduced the association between study group and health outcomes. In the final multivariable adjustment model there was only 1 (out of 11) remaining statistically significant association, which was for higher mean HADS anxiety score at 6 months in the SC group. The overall multiple correlation for RMDQ at 6 months (primary outcome) regressed on all baseline covariates was R = 0.64 (R^2^ = 0.41).

**Table 3:** Comparison of measures of health consequences between the sickness certification group (SC) and non-sickness certification group (N-SC) at 6-month follow up

	SC group n = 63	N-SC group n = 404	Mean difference (95 % CI)			
			Unadjusted	Adjusted†	Adjusted††	Adjusted†††
Roland and Morris Disability Questionnaire	7.3 (6.2)	4.2 (4.6)	3.1 (1.8, 4.4)***	2.9 (1.6, 4.2)***	0.9 (−0.3, 2.2)	0.5 (−0.8, 1.8)
Average pain—past 2 weeks	3.4 (2.6)	2.6 (2.4)	0.8 (0.2, 1.5)*	0.7 (0.1, 1.4)*	0.0 (−0.6, 0.7)	−0.3 (−1.0, 0.4)
Pain today	3.3 (2.6)	2.2 (2.5)	1.1 (0.4, 1.7)**	0.9 (0.2, 1.6)**	0.2 (−0.4, 0.9)	−0.1 (−0.8, 0.6)
Hospital Anxiety and Depression Score—anxiety	7.8 (4.4)	5.6 (4.1)	2.2 (1.0, 3.3)***	1.9 (0.8, 3.1)**	1.1 (−0.1, 2.2)	1.3 (0.2, 2.3)*
Hospital Anxiety and Depression Score—depression	6.3 (4.2)	3.8 (3.4)	2.5 (1.5, 3.5)***	2.3 (1.3, 3.3)***	1.4 (0.3, 2.4)**	0.9 (−0.1, 1.8)
Catastrophising Scale	9.0 (7.4)	7.0 (6.5)	2.0 (0.0, 4.1)*	1.3 (−0.8, 3.4)	−0.4 (−2.4, 1.7)	0.5 (−1.6, 2.5)
Tampa Scale of Kinesiophobia	40.8 (5.1)	37.1 (6.2)	3.8 (1.9, 5.6)***	3.4 (1.5, 5.3)***	1.9 (0.0, 3.8)*	1.4 (−0.3, 3.0)
Pain self-efficacy scale	36.4 (13.3)	45.8 (11.1)	−9.4 (−12.9, −5.8)***	−9.1 (−12.8, −5.4)***	−5.4 (−8.9, −1.9)**	−3.0 (−6.4, 0.5)
EQ-5D	0.69 (0.22)	0.81 (0.19)	−0.12 (−0.18, −0.07)***	−0.11 (−0.16, −0.05)***	−0.06 (−0.11, −0.00)*	−0.04 (−0.10, 0.02)
Interference with daily activities	4.5 (2.8)	3.0 (2.6)	1.5 (0.8, 2.2)***	1.5 (0.8, 2.2)***	0.6 (−0.1, 1.2)	0.1 (−0.6, 0.8)
Altered ability to work	4.6 (3.3)	2.4 (2.8)	2.1 (1.4, 2.9)***	2.1 (1.3, 2.9)***	1.2 (0.4, 1.9)**	0.5 (−0.3, 1.3)

Reference group for calculations = N-SC group

* *p* < 0.05; ** *p* < 0.01; *** *p* < 0.001

^†^ Adjusted for baseline age, gender, occupational class, GP practice code

^††^ Adjusted for baseline age, gender, occupational class, GP practice code, RMDQ (disability), pain scales

^†††^ Adjusted for baseline age, gender, occupational class, GP practice code, RMDQ (disability), pain scales, CPG (chronic pain grade), bothersomeness, EQ-5D, HADS-Anxiety, HADS-Depression, TSK (kinesiophobia), CSQ (catastrophising) and PSES (pain self-efficacy scale)

### Resource Use and Cost Estimates

A summary of health service utilisation and associated mean costs is shown in Table 4, by study group. There were more primary care consultations in the SC group than the N-SC group. Referrals to other services were similar in the two groups. Combined mean healthcare consultation costs were slightly higher in the SC group than the N-SC group (mean difference = £30.46), although the differences were not statistically significant for the ‘health care’ perspective analysis after adjustment for baseline covariates (see Table 4).

**Table 4:** Comparison of healthcare resource use (and associated costs) and productivity losses (and associated costs) between the SC and N-SC groups

	Mean no. of consultations (mean cost, £)		Mean difference in cost, £ (95 % CI)*	
	SC group	N-SC group	Unadjusted	Adjusted^†^
Primary healthcare	3.23 (80.93)	1.91 (49.33)	31.60 (19.92, 49.08)	13.98 (−1.46, 22.74)
Secondary care referrals	1.00 (50.22)	0.88 (25.18)	25.04 (−7.96, 86.34)	15.64 (−37.62, 112.01)
Referrals to private practitioners/alternative healthcare providers	0.043 (1.45)	0.058 (1.91)	−0.46 (−2.46, 2.22)	0.85 (−1.03, 2.95)

	Mean no. of working days		Mean difference in cost, £ (95 % CI)*	
	Lost (mean cost, £)		Unadjusted	Adjusted^†^
Time off work (absenteeism)	36.67 (3,185.39)	7.78 (598.14)	2,587.25 (1,743.69, 3,629.74)	1,956.06 (941.55, 3,039.99)

Aggregated (total) mean cost (£)			Mean difference in cost, £ (95 % CI)*	
			Unadjusted	Adjusted^†^
Health care perspective (primary healthcare and referrals)	132.60	76.41	56.20 (20.40, 172.53)	30.46 (−12.46, 148.28)
Societal perspective (health care perspective + time off work)	3,317.99	674.55	2,643.44 (1,688.97, 4,255.29)	1,986.53 (928.55, 3,377.04)

Healthcare consultations/referrals data was available for all 806 baseline cases. Data on absenteeism was available for a sub-sample of 439 responders to the time off work question in the 6 months follow up questionnaire

* Difference is between the SC group and the N-SC group

^†^ Adjusted for baseline age, gender, occupational class, GP practice code, RMDQ (disability), pain scales CPG (chronic pain grade), bothersomeness, EQ-5D; HADS-Anxiety; HADS-Depression; TSK (kinesiophobia); CSQ and PSES (pain self-efficacy scale)

A summary of the societal costs for the two groups is also shown in Table 4. Overall, mean societal cost was greater in the SC group compared to the N-SC group, with a mean difference of £987 (95 % CI 929, 3,377). This difference was mainly due to greater work absence in the SC group (36.7 vs. 7.8 days in the N-SC group) resulting in significantly larger productivity losses.

### Sensitivity Analysis

There were 22 individuals who reported time off work in excess of 3 months in our dataset. For the FCA analysis, these individuals had their recorded number of days off work values truncated to 60 days. Productivity and overall societal costs for this sensitivity analysis were still significantly higher: mean differences were £1485.09 and £1547.90, respectively.

## Discussion

### Summary of Findings

The findings from this study suggest that there is little difference in outcomes of pain, function and general well-being for low back pain patients who are issued a sickness certificate compared to those not issued a sickness certificate—when adjusted for imbalances in baseline characteristics. Furthermore, any difference in healthcare costs through issuing certificates is also likely to be small. Hence, from a healthcare perspective there is little to choose between issuing or not issuing sickness certificates. These data suggest that ‘sick notes are not necessarily bad for you’ and offer a reasonable support strategy for some patients. However, not unexpectedly, patients issued a sickness certificate are significantly more likely to take time off work, with negative repercussions in respect of a broader societal perspective that also considers the importance of higher work absenteeism and greater overall societal cost.

There is clear evidence from our data that patients issued sickness certificates are different to those who are not issued certificates. The former were more likely to be male and have manual occupations. Patients issued sick certificates had significantly greater severity of pain and psychosocial obstacles to recovery at baseline. Upon crude (unadjusted) review of 6-month follow up health outcomes, patients issued sickness certificates still had significantly worse pain, psychosocial and general health outcomes. However, through sequentially adjusting for the differences in baseline characteristics we were able to identify differences in baseline levels of pain and disability as key reasons for the difference in 6-month outcomes between study groups. One significant association remained in the fully adjusted model—the association with HADS anxiety. This needs to be viewed cautiously given the observational design of our study and the heightened issue of multiplicity given the fact that several statistical tests were performed. The covariates included in the (adjusted) outcome model cover a range of baseline demographic and clinical factors that are known to be prognostic of outcome—and therefore of interest as potential confounders in the relationship between study group and clinical outcomes. Other (unmeasured) variables, notably work-specific variables may well explain additional variance though could not be accounted for in estimating the true study group difference. Unmeasurable factors may also play a part. Together, these unmeasured factors could potentially explain the residual difference between study groups in respect of health outcomes at 6 months.

There were differences in costs—most notably costs related to productivity loss through absenteeism. For the main analysis, productivity costs were calculated using the most commonly used approach—the human capital approach (HCP), which assumes the cost is lost throughout the full period of absenteeism. An alternative approach to this is the friction cost approach (FCA) whereby long-term absentee losses have a fixed horizon (which denotes time it takes to replace the skills of the absentee). Productivity costs for these individuals are applicable up to the ‘time of replacement’ with 3 months a justifiable friction period [46]. We assessed the robustness of the findings to the difference in evaluative approach and found consistent results showing significantly higher societal costs for patients issued sickness certificates.

### Strengths and Limitations

This is the first study to investigate outcome and cost consequences of sickness certification in a primary care population with low back pain. It was based on a large cohort of primary care consulters; low back pain is most commonly managed in primary care. About 98 % of patients in the UK are registered with an NHS GP [47]. Linking questionnaire data with electronic medical record downloads allowed for examination of patients’ self-reported clinical status within the SC and N-SC groups. The medical record data have been verified to be accurate [33], ensuring that sickness certification is measured objectively, thus reducing the potential for recall and ascertainment bias. Key prognostic variables were collected at baseline making it possible to carry out an adjusted comparison of the two study groups.

However, there were several limitations. The data were collected about 8–10 years ago and there have since been important UK developments, including changes to the sick certificate (now referred to as ‘fit note’), in improving primary care management of patients with back pain affecting their work ability since the time of the study. There was large attrition—though this was fairly similar in both study groups. Also, responder/non-responder baseline clinical characteristics were similar—so there was little concern regarding imbalances in important prognostic variables. Both these issues imply that an analysis based on complete cases would not yield biased between-group comparisons [48]. Employment questions did not distinguish full-time and part-time work; so we calculated all costs on the basis of full-time work. Job satisfaction was assessed, but no other measures of physical job demands or organizational support were assessed; these factors are also likely to have a major role in the decision-making of clinicians on issues of sickness certification. Hospital admissions and/or length of stay (which are important cost drivers) [49], were not readily accessible from the electronic GP databases, though hospital admission is a rare occurrence in back pain patients so the impact will be limited [50]. Medical record data do not provide information about the duration of each certificate; the most common duration is reported to be 10 working days or 2 weeks [51–54], and this was the interval we ascribed to each certificate in our study. A further limitation is that we cannot be sure that the issued sickness certificate was for the episode of back pain that the patients consulted with. A previous study, using the same methods as in our study, matched self-reported absence for low back pain with sickness certificates in the electronic medical records and found that 95 % of records matched [33].

### Comparison with Existing Literature

The significant baseline differences in pain, disability, anxiety, depression, fear of movement, and pain self-efficacy between those who did and did not receive a sickness certificate suggest that those receiving certificates are the more complex patients who present with greater pain and disability as well as greater related psychological distress. Morris and Watson [55] also found that patients who receive sickness certificates reported significantly more pain, perceived disability and fear avoidance beliefs about work than those not receiving sickness certificates. Patients with common mental health problems, such as anxiety, consult more frequently and receive more sickness certificates than those without common mental health problems [56], and the rates of certification in the UK confirm this [57].

### Implications for Practice

There are clear implications not just in terms of the clinical time but also the skills required to manage complex patients in the return to work process. GPs frequently report that they feel under-skilled in managing occupational health issues [51, 58], despite current courses from the Royal College of General Practitioners [59], and advice aimed specifically at GPs and patients [60, 61]. Furthermore, it is unclear to what extent issuing a sickness certificate is helpful to patients in managing their episode of back pain. It is possible that certification contributes to a cycle of disability for some patients, such as that represented by the fear avoidance model [62], reinforcing the unhelpful message that patients with back pain should avoid their usual activities.

On the other hand, issuing of a sickness certification may be viewed as a token for providing considerate, patient-centred care. In this context, emphasis is on expression of empathy and compassionate care, to potentially reduce unnecessary suffering, or symptom exacerbation, or to avoid embarrassment and stigmatization at work. For example, for a patient who is barely able to sit or stand comfortably, who is experiencing a high level of emotional distress, with an uncooperative employer and few social supports and coping resources, it may seem inhumane for the GP to recommend immediate return to work. Also, against the backdrop of encouraged return to work, is the unknown quantity of how going back to work affects an individuals’ work performance and his/her interactions with co-workers and employers. Contrasting ethical/moral issues against clear societal productivity and cost losses is a challenge faced by clinicians and policymakers in making difficult decisions about the care of low back pain patients.

The new ‘fit note’ (issued in the UK in 2010) is an adaptation of the traditional sick-note to help encourage and mediate the process of return-to-work with emphasis on doctor assessment and provision of recommendation regarding the patients’ capacity (and extent) to work, whether this may be “not fit for work” or “may be fit for work” under certain specified restrictions that should be taken into account by the employer. This gives the GP more options than the traditional sickness certification whereby ‘sick leave’ was dichotomous i.e. sick leave or not. Through fit notes, GPs record details of the functional effects of their patient’s condition so that patients and their employers can consider ways to help the patient return to work. In this way, GPs can effectively manage patients’ expectations about their capability for work; give a clear clinical assessment to guide the patient about the impact of their condition on their fitness for work; help sustain relations between the patient and employer by allowing communication to take place around work adaptation and support at work. In all, the revised ‘fit-note’ certification offers greater flexibility and could help improve clinical outcome as well as potentially reducing the burden of lost productivity and societal cost.

Our findings have shown that GPs’ issuing of sickness certificates is unlikely to be associated with positive cost consequences, particularly from a societal perspective. After adjusting for several potential baseline confounding factors, sick certification on average resulted in higher healthcare costs and significantly greater costs related to work absenteeism. It is the difference in productivity loss between the two study groups that generated the greatest differences in cost. This is not a surprising finding because the issuing of a sickness certificate will in itself result in about 10 days lost from work [51–54]. If all participants who were given a sick certificate were to then recover and go back to work without any further problems, the expected total number of absent days would be about 10 days. However, the observed figure was approximately three times that amount, and the disparity between the SC group and the N-SC group in terms of days absent from work was considerably greater than the 10 days advocated in the original certificate.

In conclusion, this study has provided no clear evidence that issuing of sickness certificates confers any major advantages or disadvantages in respect of patient improvements in clinical outcomes or healthcare costs. However, from a broader societal perspective, primary care clinicians and policymakers may wish to consider the higher societal costs as well as the ethical/moral issues of sickness certification within patient care. In general, primary care providers need more treatment options and resources for managing back disability (other than writing sickness certificates).
